# Supplementary material for: Selective electroreduction of carbon dioxide to methanol on copper selenide nanocatalysts
Source: Nat Commun. 2019 Feb 8;10:677. doi: 10.1038/s41467-019-08653-9 (PMC6368552; doi:10.1038/s41467-019-08653-9)
Supplement: Supplementary file 1 — Supplementary Information [file 41467_2019_8653_MOESM1_ESM.pdf]

Supplementary Information

Selective Electrocatalytic Carbon Dioxide Reduction to Methanol on Copper Selenide Nanocatalysts

Yang et al.

Supplementary Figures

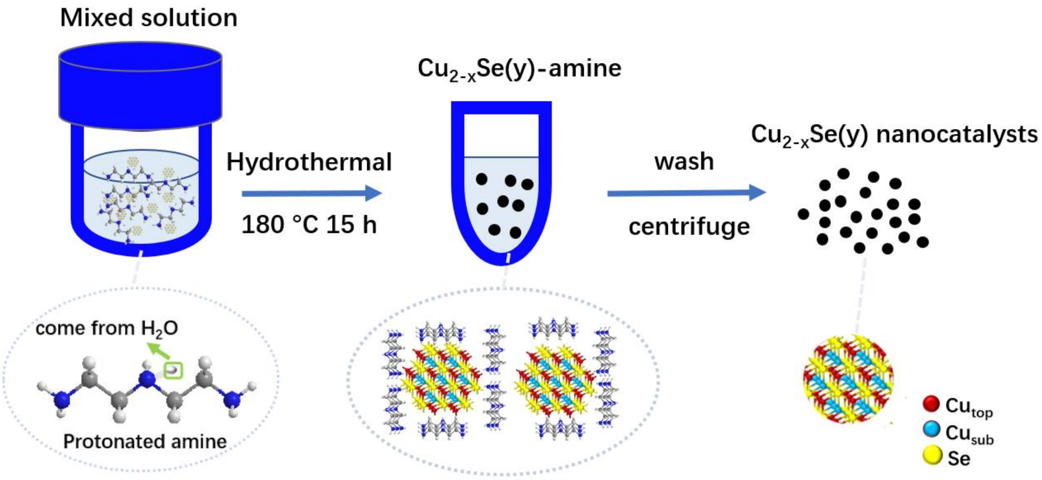

Supplementary Fig. 1. The route for the synthesis of $Cu_{2-x}Se(y)$ nanocatalysts.

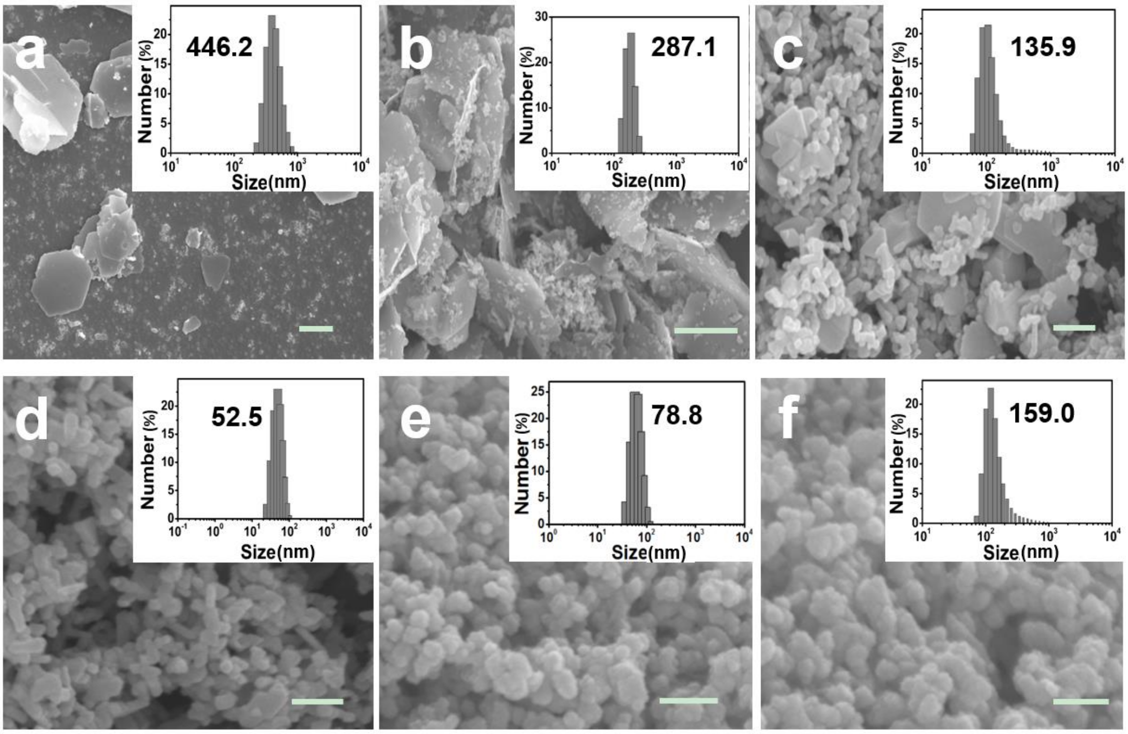

Supplementary Fig. 2. SEM and DLS images of various $Cu_{2-x}Se$(y) nanocatalysts. (a) $Cu_{1.61}Se$(1/0); (b) $Cu_{1.60}Se$(3/1); (c) $Cu_{1.63}Se$(1/1); (d) $Cu_{1.63}Se$(1/3); (e) $Cu_{1.62}Se$(1/5); (f) $Cu_{1.64}Se$(0/1). Scale bar = 200 nm.

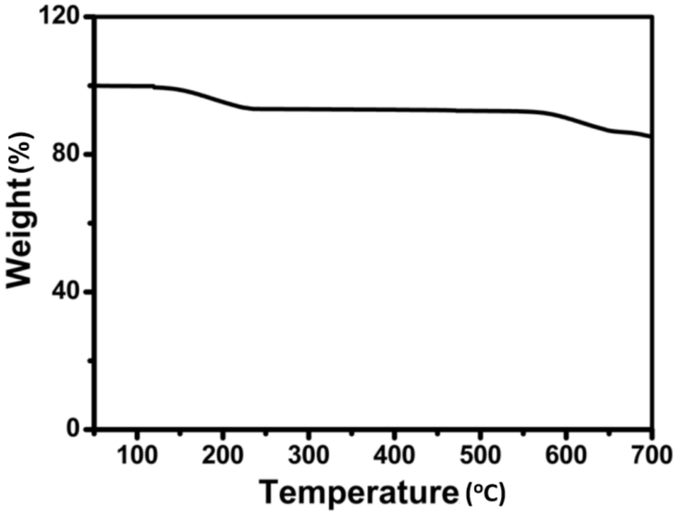

Supplementary Fig. 3. TG curve of $Cu_{1.63}Se$(1/3) nanocatalyst.

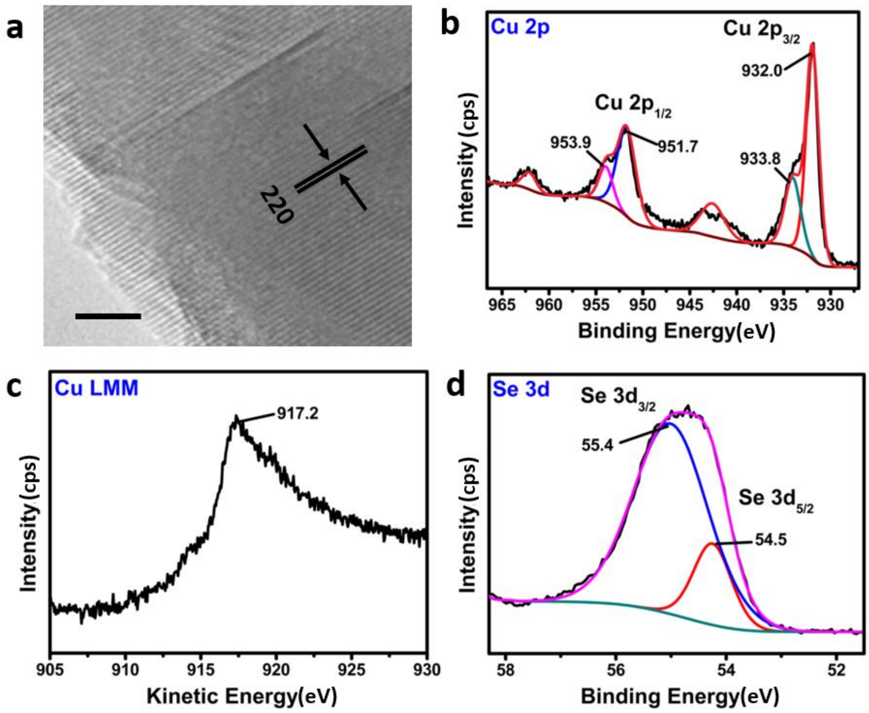

Supplementary Fig. 4. Characterization of $Cu_{1.61}Se$(1/0) nanocatalysts. (a) HR-TEM image of $Cu_{1.61}Se$(1/0) nanocatalysts, scale bar = 5 nm; XPS spectra of $Cu_{1.61}Se$(1/0) nanocatalysts: (b) Cu 2p; (c) Cu LMM and (d) Se 3d.

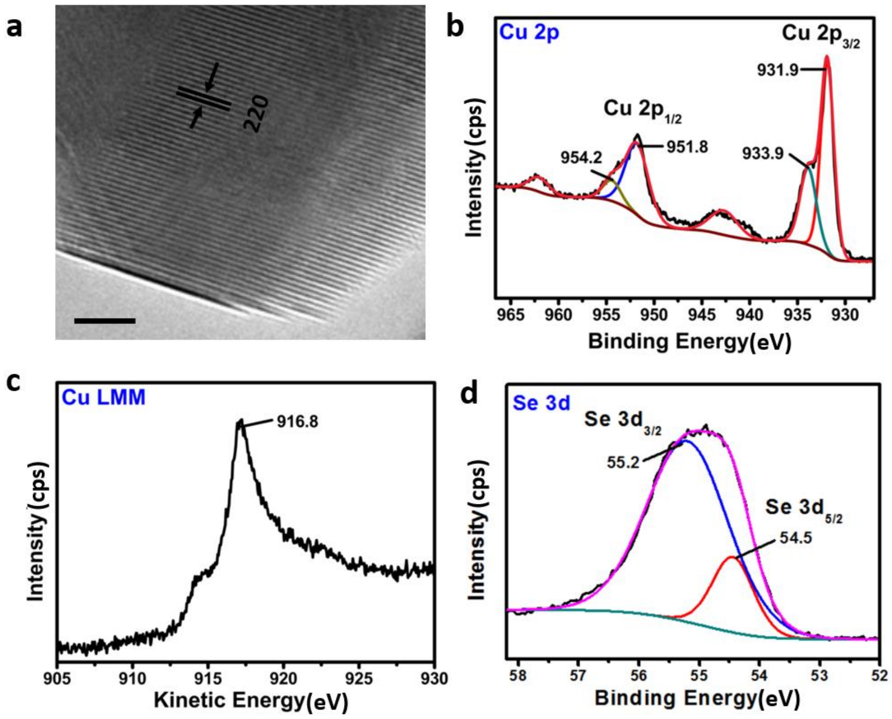

Supplementary Fig. 5. Characterization of $Cu_{1.60}Se$(3/1) nanocatalysts. (a) HR-TEM image of $Cu_{1.60}Se$(3/1) nanocatalysts, scale bar = 5 nm; XPS spectra of $Cu_{1.60}Se$(3/1) nanocatalysts: (b) Cu 2p; (c) Cu LMM and (d) Se 3d.

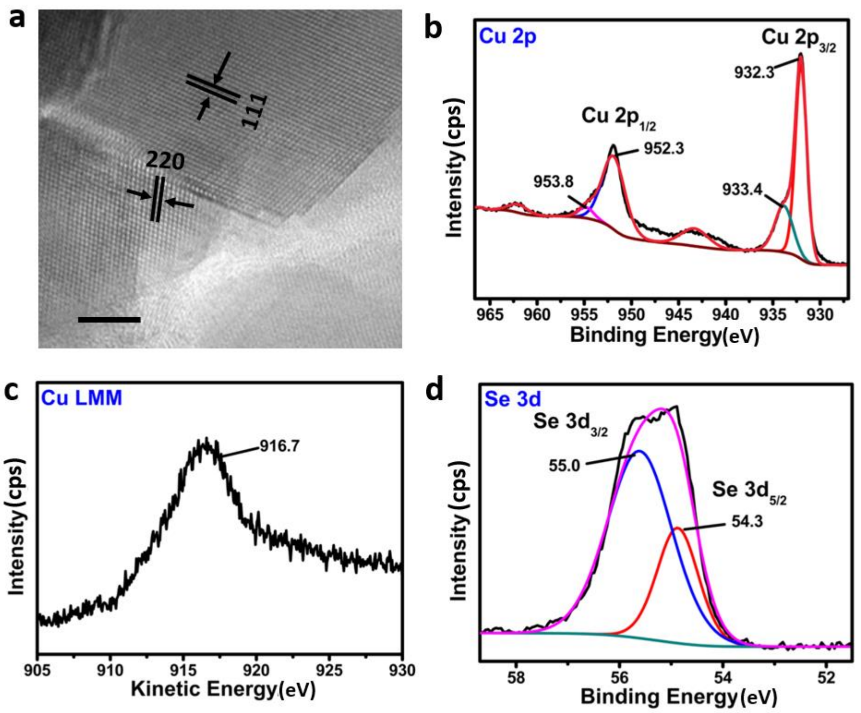

Supplementary Fig. 6. Characterization of $Cu_{1.63}Se$(1/1) nanocatalysts. (a) HR-TEM image of $Cu_{1.63}Se$(1/1) nanocatalysts, scale bar = 5 nm; XPS spectra of $Cu_{1.63}Se$(1/1) nanocatalysts: (b) Cu 2p; (c) Cu LMM and (d) Se 3d.

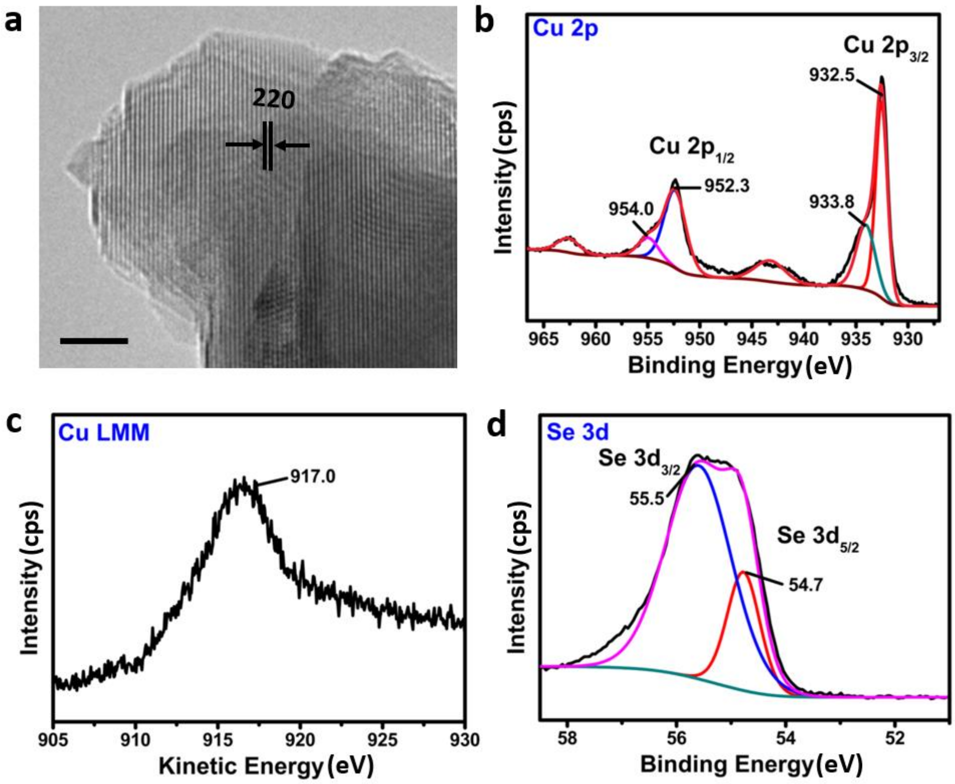

Supplementary Fig. 7. Characterization of $Cu_{1.62}Se$(1/5) nanocatalysts. (a) HR-TEM image of $Cu_{1.62}Se$(1/5) nanocatalysts, scale bar = 5 nm; XPS spectra of $Cu_{1.62}Se$(1/5) nanocatalysts: (b) Cu 2p; (c) Cu LMM and (d) Se 3d.

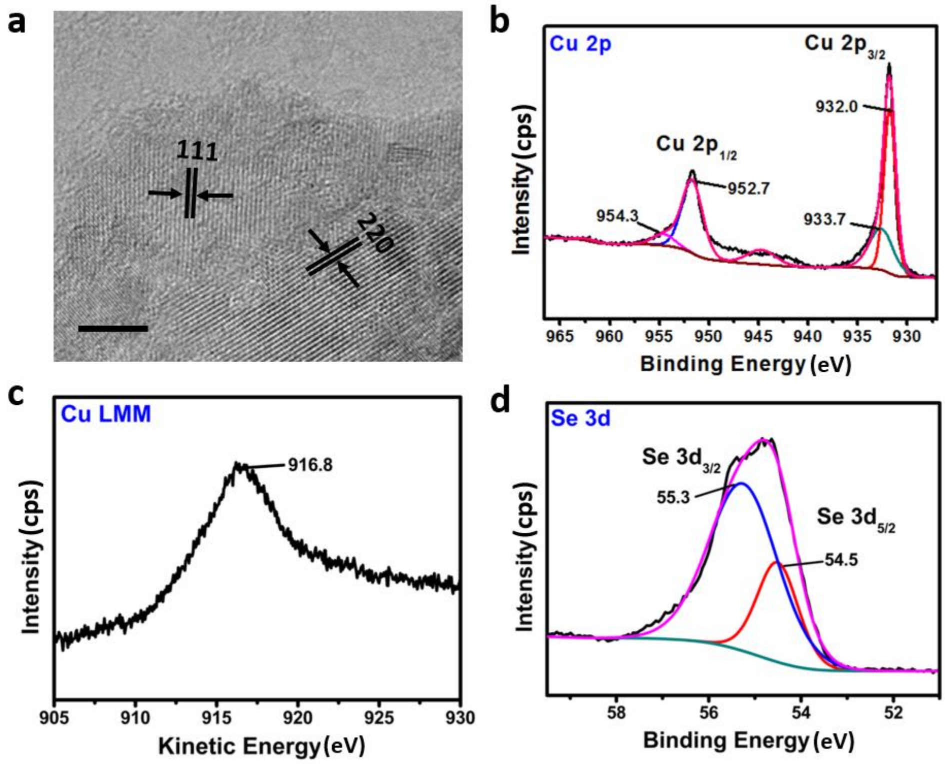

Supplementary Fig. 8. Characterization of $Cu_{1.64}Se(0/1)$ nanocatalysts. (a) HR-TEM image of $Cu_{1.64}Se(0/1)$ nanocatalysts, scale bar = 5 nm; XPS spectra of $Cu_{1.64}Se(0/1)$ nanocatalysts: (b) Cu 2p; (c) Cu LMM and (d) Se 3d.

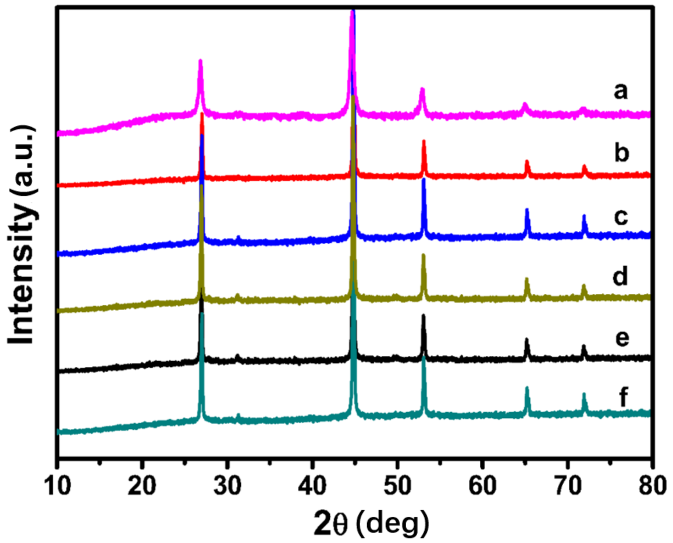

Supplementary Fig. 9. XRD patterns of various $Cu_{2-x}Se$(y) nanocatalysts. (a) $Cu_{1.61}Se$(1/0); (b) $Cu_{1.60}Se$(3/1); (c) $Cu_{1.63}Se$(1/1); (d) $Cu_{1.63}Se$(1/3); (e) $Cu_{1.62}Se$(1/5); (f) $Cu_{1.64}Se$(0/1).

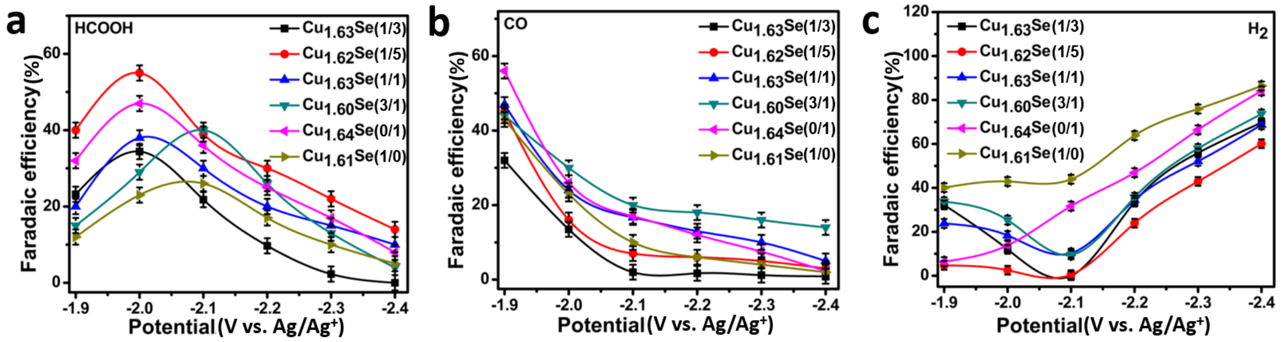

Supplementary Fig. 10. Electrolysis results. FE of various products over different electrodes at different applied potentials: (a) HCOOH; (b) CO; (c) H_2. All data are presented as mean ± s. d.

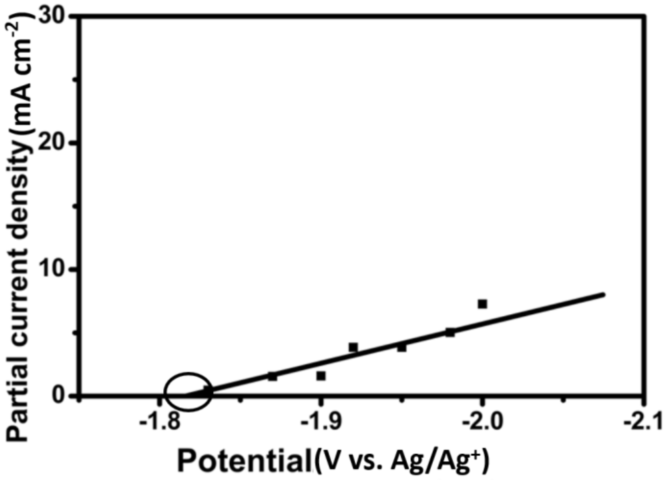

Supplementary Fig. 11. Partial current densities of methanol under different potentials. The equilibrium potential can be obtained by extrapolation zero partial current density.

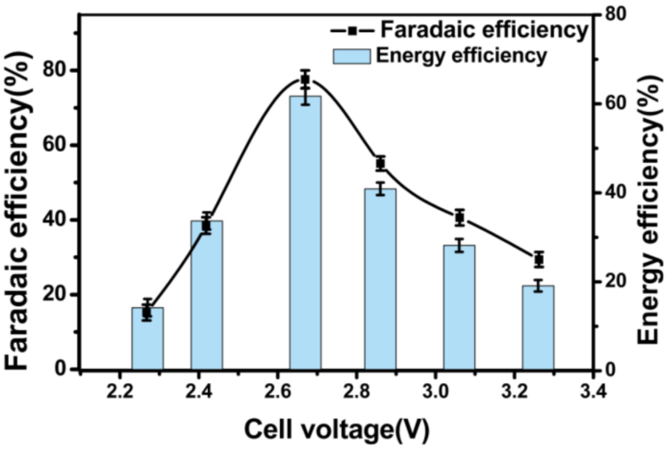

Supplementary Fig. 12. The dependence of FE for methanol and energy efficiency of the CO_2 electroreduction to methanol on applied cell voltage. All data are presented as mean ± s. d.

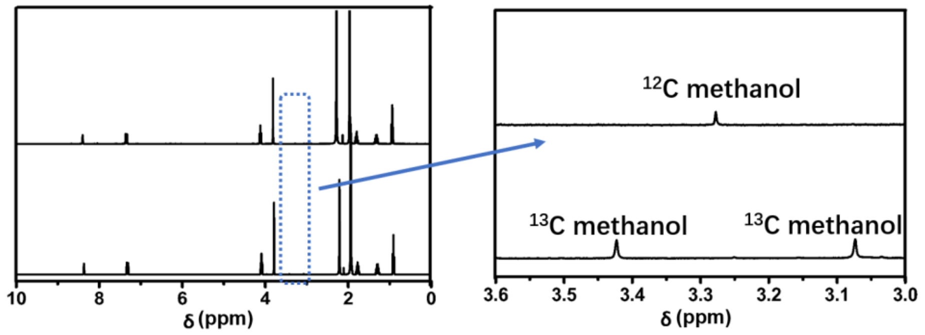

Supplementary Fig. 13. ^{1}H-NMR spectrum of [Bmim]PF_6 (30 wt%)/CH_3CN/H_2O (5 wt%) electrolyte after 5 h electrolysis on $Cu_{1.63}Se$(1/3) electrode in $^{12}CO_2$-saturated and $^{13}CO_2$-saturated solutions.

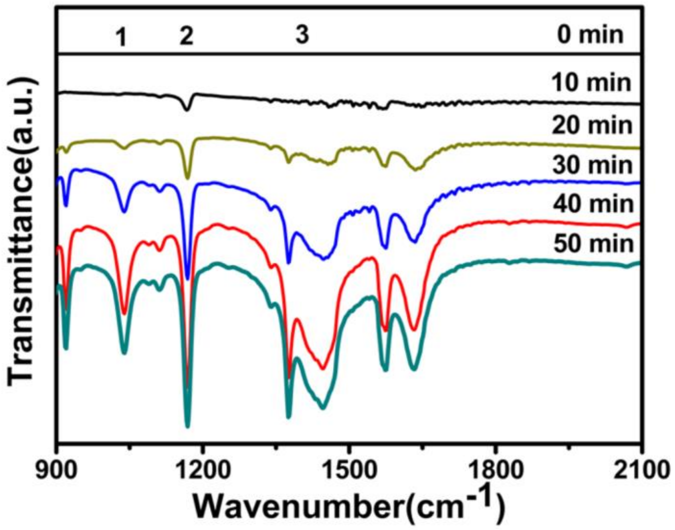

Supplementary Fig. 14. IR spectra of the electrolyte phase over $Cu_{1.63}Se$(1/3) electrode in [Bmim]PF_6 (30 wt%)/CH_3CN/H_2O (5 wt%) electrolyte at different electrolysis times and -2.1 V vs. Ag/Ag^+. The background used was the signal of the original CO_2-saturated electrolyte. The intensity of the characteristic absorption bands at 1085 cm^{-1}, 1140 cm^{-1} and 1420 cm^{-1} can be assigned to C-O (1), C-H (2) and CH_3 (3), and the intensity increased with increasing electrolysis time, indicating that the amount of methnaol generated increased with electrolysis time.

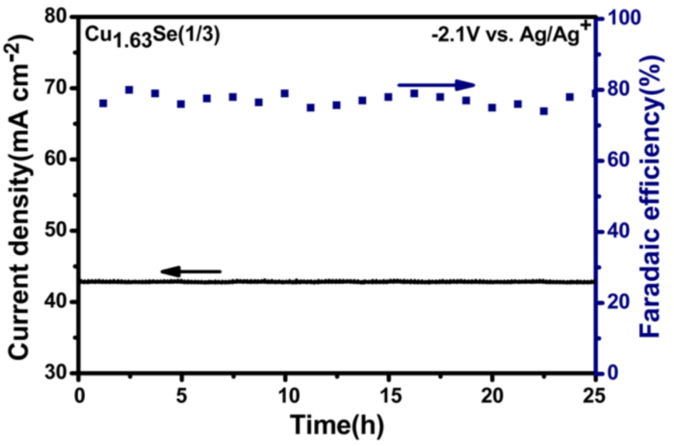

Supplementary Fig. 15. The long-term stability of the $Cu_{1.63}Se(1/3)$ catalyst at the applied potentials of −2.1 V vs. Ag/Ag^{+}.

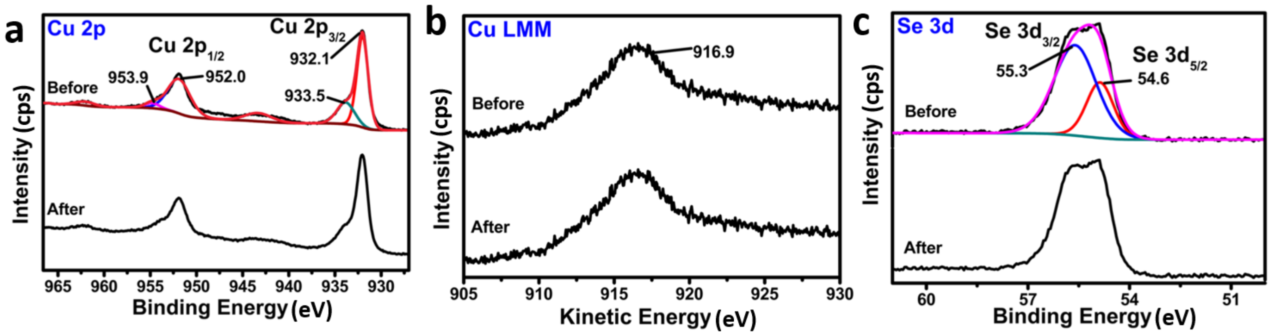

Supplementary Fig. 16. XPS spectra of $Cu_{1.63}Se(1/3)$ before and after 25 h electrolysis. (a) Cu 2p; (b) Cu LMM 2p and (c) Se 3d.

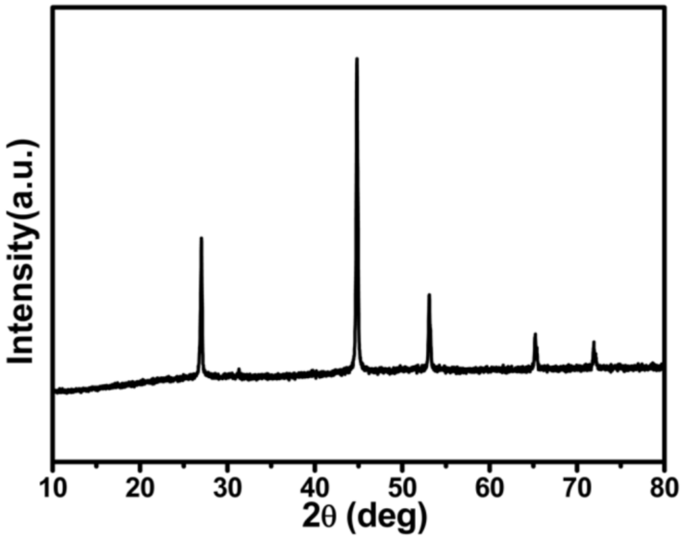

Supplementary Fig. 17. XRD pattern of $Cu_{1.63}Se$(1/3) after electrolysis of 25 h.

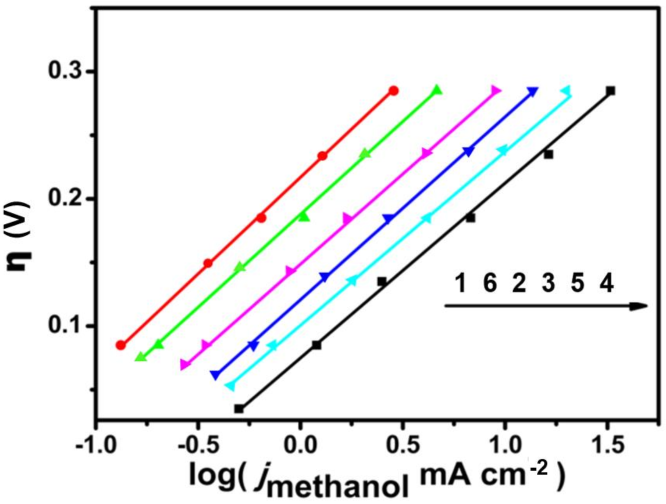

Supplementary Fig. 18. Tafel plot for CH_3OH production over various $Cu_{2-x}Se$(y) nanocatalysts: 1) $Cu_{1.61}Se$(1/0); 2) $Cu_{1.60}Se$(3/1); 3) $Cu_{1.63}Se$(1/1); 4) $Cu_{1.63}Se$(1/3); 5) $Cu_{1.62}Se$(1/5) and 6) $Cu_{1.64}Se$(0/1).

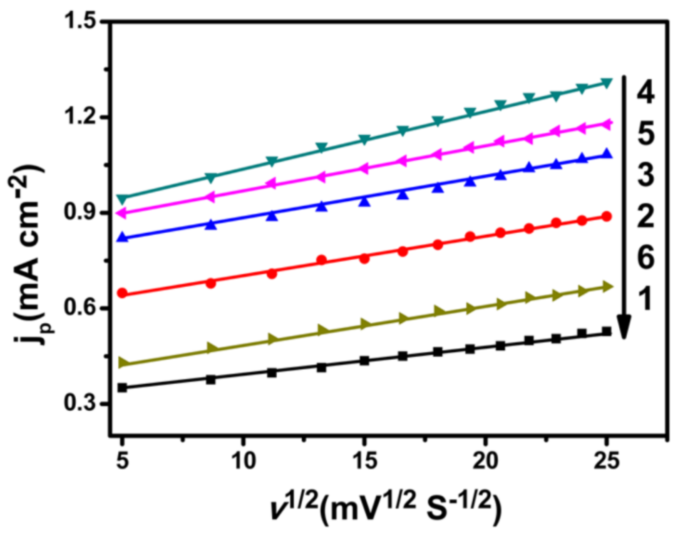

Supplementary Fig. 19. Charging current density against scan rates (*v*) over different electrodes in CO_2-saturated [Bmim]PF_6 (30 wt%)/CH_3CN/H_2O (5 wt%) ternary electrolyte. (1) $Cu_{1.61}Se$(1/0); (2) $Cu_{1.60}Se$(3/1); (3) $Cu_{1.63}Se$(1/1); (4) $Cu_{1.63}Se$(1/3); (5) $Cu_{1.62}Se$(1/5) and (6) $Cu_{1.64}Se$(0/1).

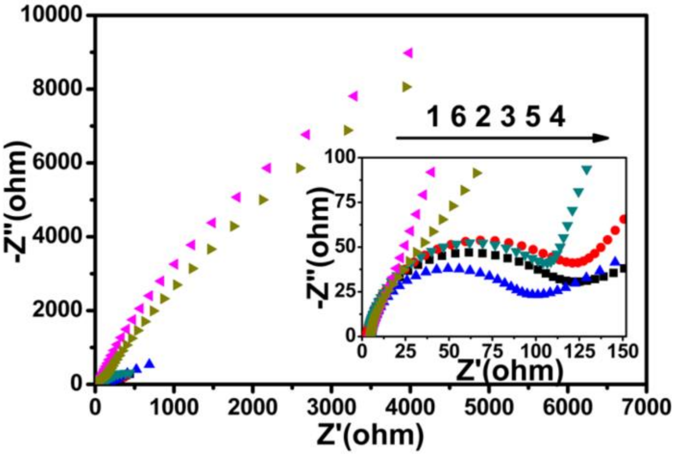

Supplementary Fig. 20. The experimental and simulated EIS spectra of various electrodes in CO_2-saturated [Bmim]PF_6 (30 wt%)/CH_3CN/H_2O (5 wt%) ternary electrolyte. (1) $Cu_{1.61}Se$(1/0); (2) $Cu_{1.60}Se$(3/1); (3) $Cu_{1.63}Se$(1/1); (4) $Cu_{1.63}Se$(1/3); (5) $Cu_{1.62}Se$(1/5) and (6) $Cu_{1.64}Se$(0/1).

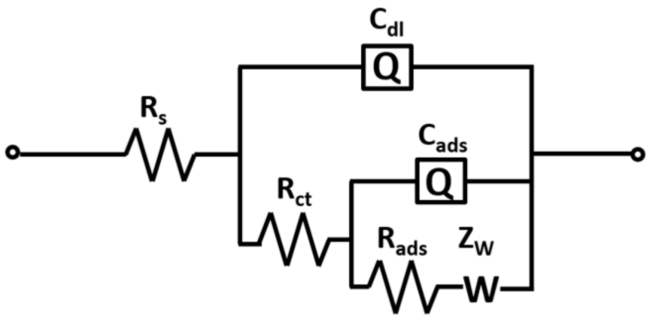

Supplementary Fig. 21. Electrical equivalent circuit used for simulating the experimental impedance data. The components contain solution resistance (R_s), electron transfer resistance (R_{ct}), double layer capacitance (C_{dl}), surface adsorption capacitance (C_{ads}), surface adsorption resistance (R_{ads}) and Warburg-type impedance (Z_w).

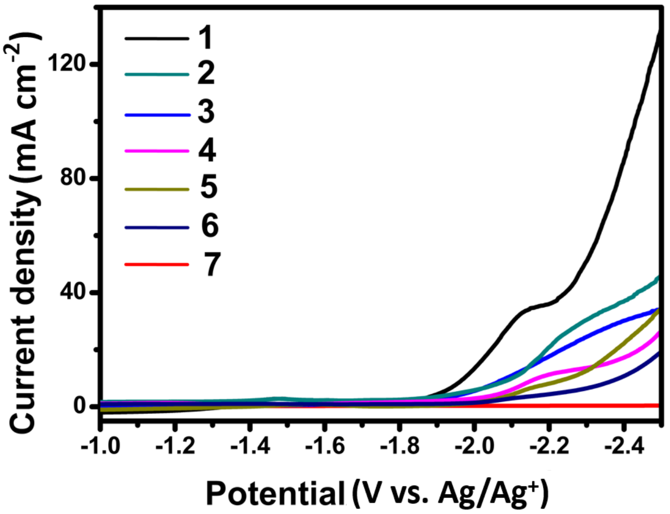

Supplementary Fig. 22. LSV traces recorded using $Cu_{1.63}Se$(1/3) electrodes in various electrolytes. (1) [Bmim]PF_6 (30 wt%)/CH_3CN/H_2O (5 wt%); (2) [Bmim]TF_2N (30 wt%)/CH_3CN/H_2O (5 wt%); (3) [Bmim]BF_4 (30 wt%)/CH_3CN/H_2O (5 wt%); (4) [Bmim]OAc (30 wt%)/CH_3CN/H_2O (5 wt%); (5) [Bmim]NO_3 (30 wt%)/CH_3CN/H_2O (5 wt%); (6) [Bmim]ClO_4 (30 wt%)/CH_3CN/H_2O (5 wt%) and (7) 0.5 M $TEAPF_6$ in the CH_3CN (92.86 wt%) and H_2O (7.14 wt%) mixture solution.

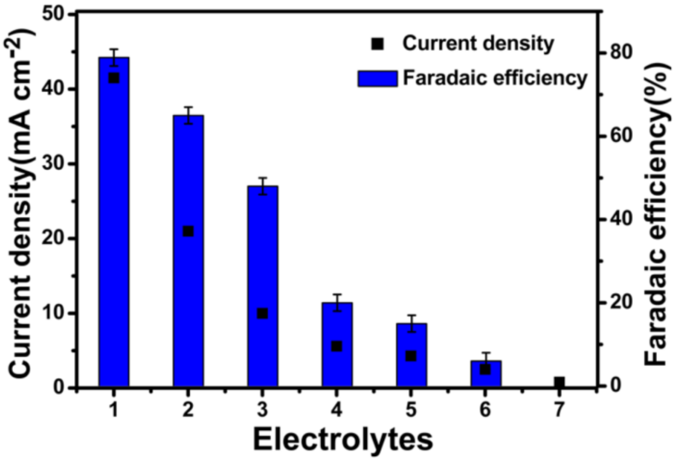

Supplementary Fig. 23. Current density and Faradaic efficiency of $Cu_{1.63}Se$(1/3) electrodes for electroreduction CO_2 to methnaol in various electrolytes at −2.1 V vs. Ag/Ag^+. (1) [Bmim]PF_6 (30 wt%)/CH_3CN/H_2O (5 wt%); (2) [Bmim]TF_2N (30 wt%)/CH_3CN/H_2O (5 wt%); (3) [Bmim]BF_4 (30 wt%)/CH_3CN/H_2O (5 wt%); (4) [Bmim]OAc (30 wt%)/CH_3CN/H_2O (5 wt%); (5) [Bmim]NO_3 (30 wt%)/CH_3CN/H_2O (5 wt%); (6) [Bmim]ClO_4 (30 wt%)/CH_3CN/H_2O (5 wt%) and (7) 0.5 M $TEAPF_6$ in the CH_3CN (92.86 wt%) and H_2O (7.14 wt%) mixture solution. All data are presented as mean ± s. d.

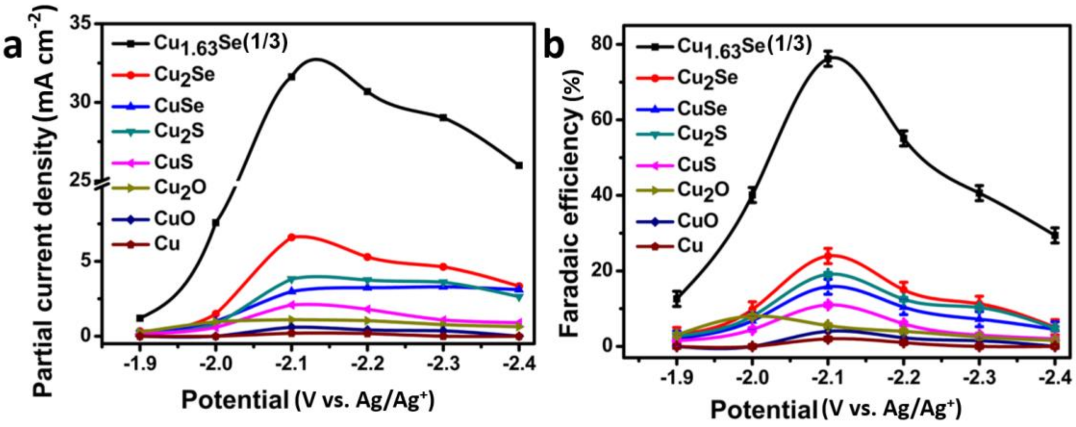

Supplementary Fig. 24. Electrolysis results over various catalysts. (a) Partial current density and (b) FE of methanol on various electrodes at different applied potentials. All data in b are presented as mean ± s. d.

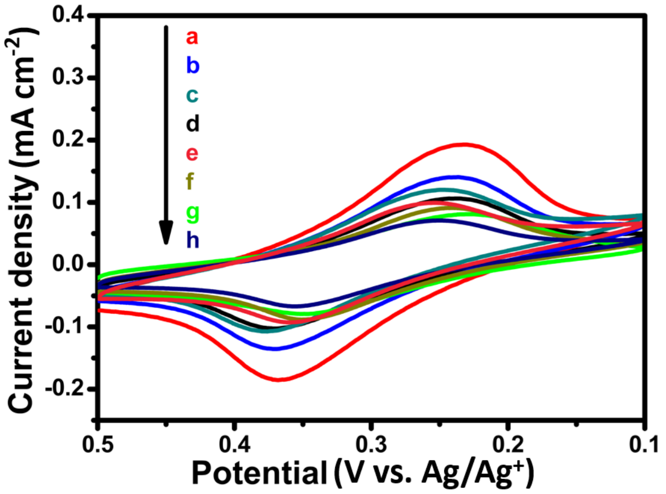

Supplementary Fig. 25. Cyclic voltammetry curves over various catalysts in 5 mM $K_4Fe(CN)_6$/0.1 M KCl solution with scan rate of 5 mV s^{-1}. (a) $Cu_{1.63}Se$(1/3); (b) Cu_2Se; (c) Cu_2S; (d) CuSe; (e) CuS; (f) Cu_2O; (g) CuO and (h) Cu.

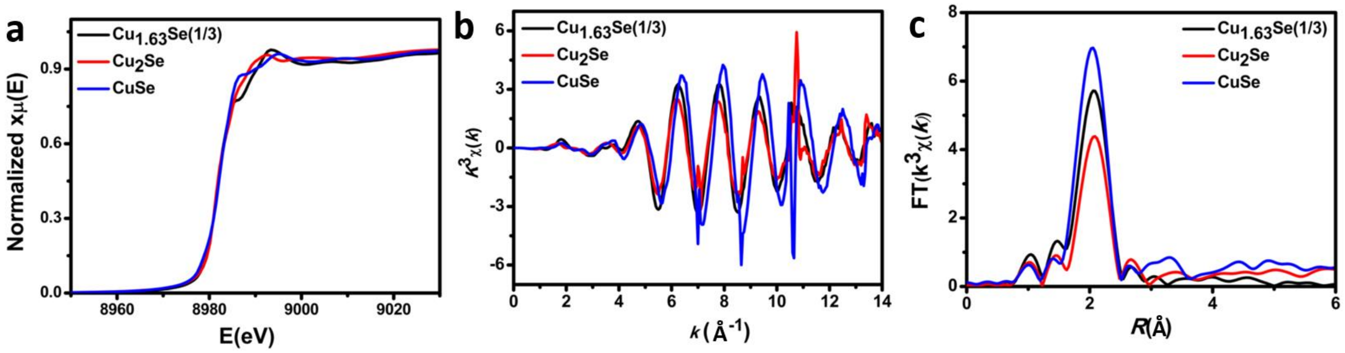

Supplementary Fig. 26. EXAFS results. (a) Normalized Cu K-XANES spectra; (b) Cu K-edge extended EXAFS oscillation function $k^2\chi(k)$; (c) the corresponding Fourier transforms $FT(k^3\chi(k))$ for $Cu_{1.63}Se$(1/3), Cu_2Se and CuSe.

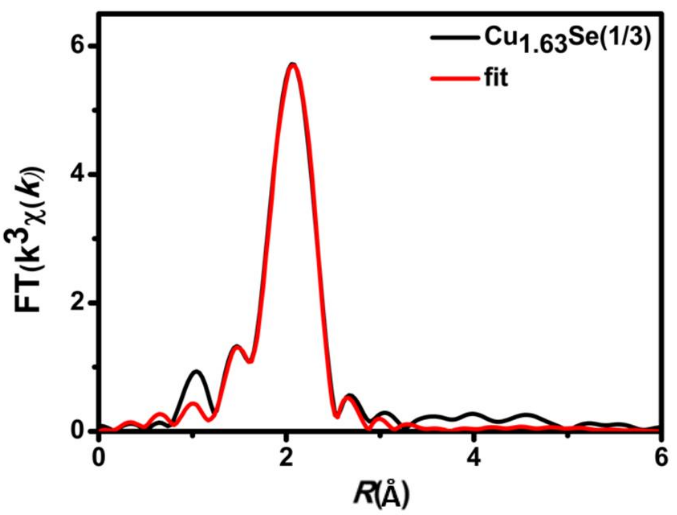

Supplementary Fig. 27. The EXAFS fitting results of the Cu K-edge for $Cu_{1.63}Se(1/3)$.

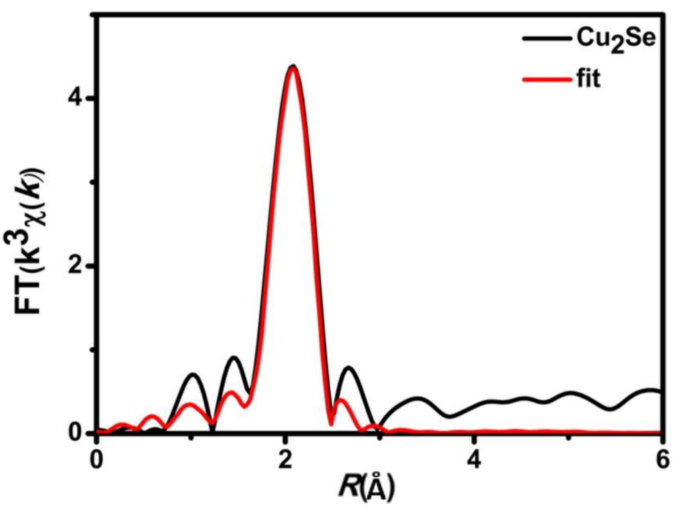

Supplementary Fig. 28. The EXAFS fitting results of the Cu K-edge for Cu_2Se.

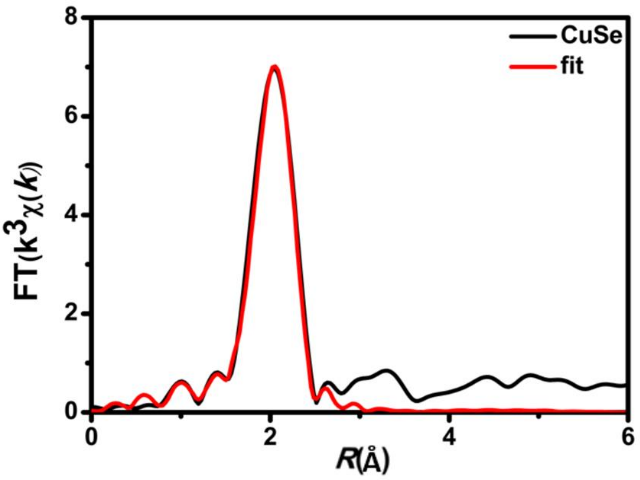

Supplementary Fig. 29. The EXAFS fitting results of the Cu K-edge for CuSe.

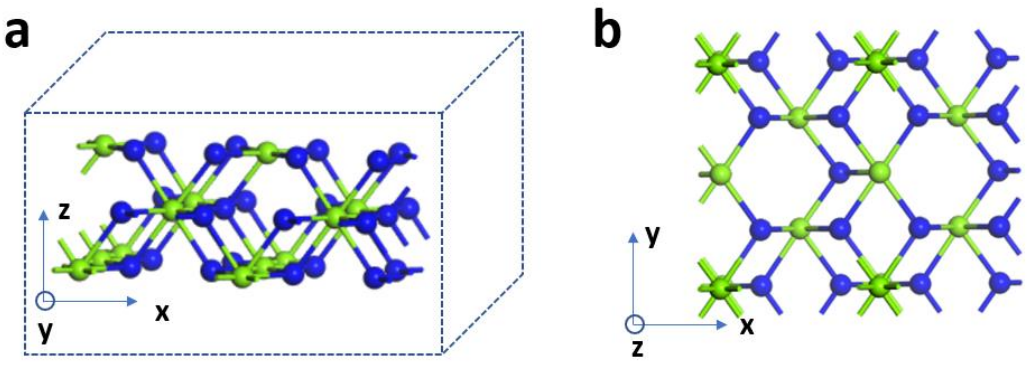

Supplementary Fig. 30. Crystal structure of $Cu_{1.63}Se$(1/3): (a) view from y axis; (b) view from z axis. The atoms in blue and green represent Cu and Se.

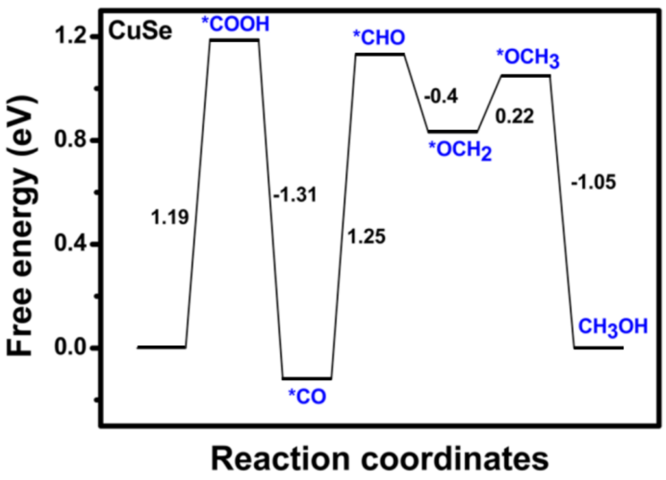

Supplementary Fig. 31. Free energy diagrams for CO_2 electroreduction to methanol on CuSe electode.

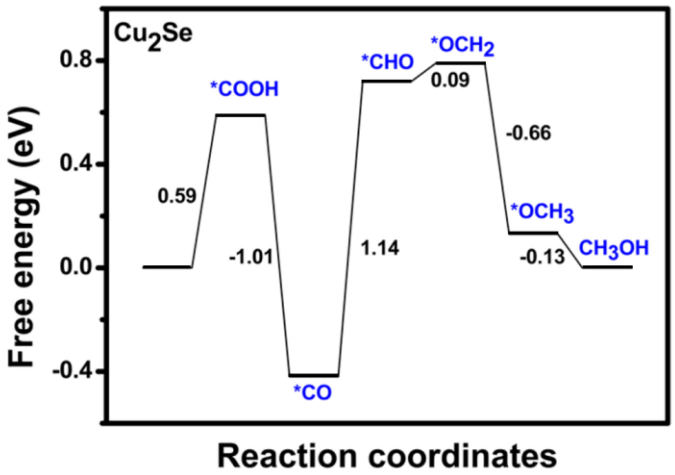

Supplementary Fig. 32. Free energy diagrams for CO_2 electroreduction to methanol on Cu_2Se electode.

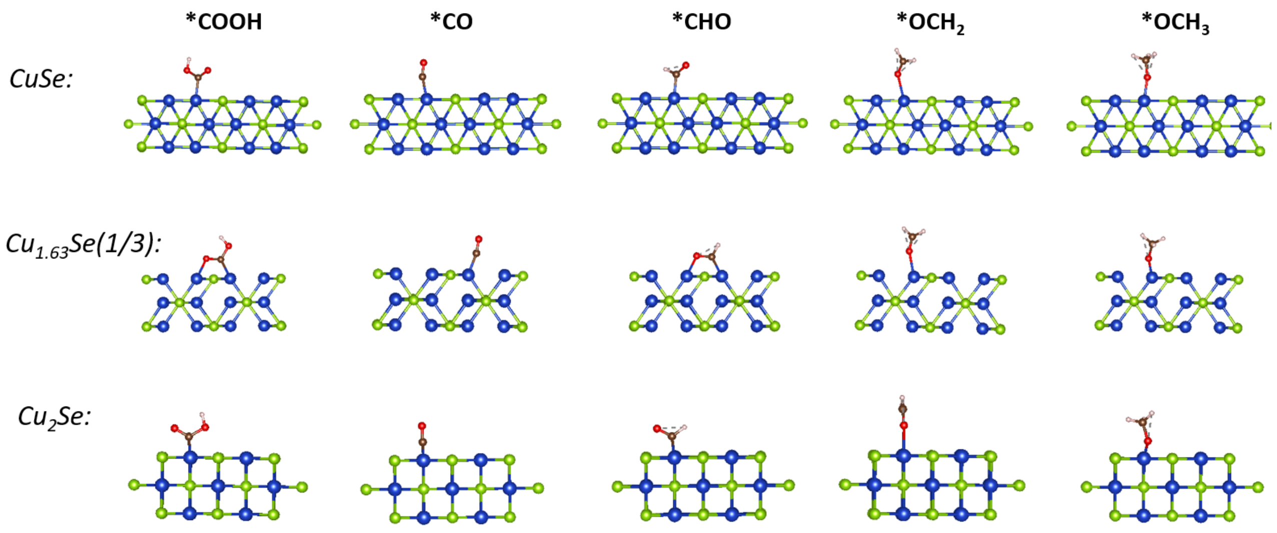

Supplementary Fig. 33. Side view of the adsorption configurations of reaction intermediates on the three simulated surfaces. The atoms in blue, green, brown, red, and pink represent Cu, Se, C, O, and H, respectively.

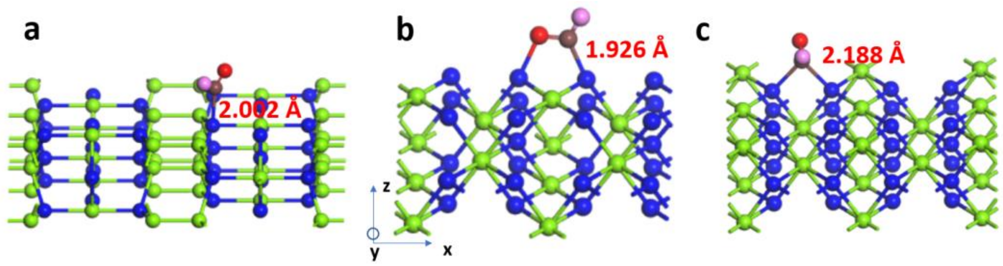

Supplementary Fig. 34. Optimized geometries of the CHO on various catalysts. (a) CuSe, (b) $Cu_{1.63}Se(1/3)$ and (c) Cu_2Se. Bond lengths in a-c are in Å. The atoms in blue, green, brown, red, and pink represent Cu, Se, C, O, and H, respectively.

Supplementary Tables

Table 1. Electrochemical reduction of CO_2 to methanol using various electrodes and electrolytes.

Electrode/ electrocatalysts	Electrode potential (V)	Electrolyte	j_{tot}^{a} (mA cm^{-2})	$FE^{b}_{methanol}$ (%)	References
$Cu_{1.63}Se$(1/3)	-2.1 V vs. Ag/Ag$^+$	[Bmim]PF_6 (30 wt%)/CH_3CN/H_2O (5 wt%)	41.5	77.6±2.0	This work
[PYD]@Pd composite	-0.6 V vs. SCE	0.5 M KCl (aq)	~45	35	1
Pd/SnO_2	-0.24 vs. RHE	0.1M $NaHCO_3$	~1.5	54.8	2
[PYD]@Cu-Pt[c]	-0.6 V vs. SCE	0.5 MKCl	-	37	3
Pd or Pt/pyridinium	-	0.5 M KCl (aq)	0.05	22 ± 2	4
$Pd_{83}Cu_{17}$	-2.1 V vs. Ag/Ag$^+$	25 mol% [Bmim]BF_4 and 75 mol% water	31.8	80	5
Pt meshes	-0.55 V vs. Ag/AgCl	10 mM pyridoxine, 0.1 M KCl	-	~5	6
Pt disc	-0.75 V vs. Fc$^{0/+}$	0.1 M $LiClO_4$ in CH_3CN/H_2O (40% v/v)	-	36	7
RuO_2-TiO_2 nanoparticle[d]	-0.8 V vs. SCE	0.5 M $NaHCO_3$	-	< 60	8
Ru/Cu	-0.8 V vs. SCE	0.5 M $NaHCO_3$	-	41.3	9
Cu_2O-MWCNTs[e]	-0.8 V vs. Ag/AgCl	0.5 M $NaHCO_3$	-	38	10
Cu_2O/ZnO-based electrodes	-1.3 V vs. Ag/AgCl	0.5 M $KHCO_3$	10.64	17.7	11
Cu (88) Sn (6) Pb (6) alloy	-0.6 V vs. Ag/AgCl	1.5M HCl-0.17M $BaCl_2$	0.41	36.3	12
Cu nanocluster-ZnO (1010)	-1.4 V vs. Ag/AgCl	0.1M $KHCO_3$	12	2.8	13
Cu(I) oxide	-1.1 V vs. SCE	0.5 M $NaHCO_3$	-	38	14
HKUST-1[f]	-0.9V vs.	0.5 M $KHCO_3$	10.83	5.6	15

	Ag/AgCl				
Mo-Bi BMC nanosheet[g]	-0.7 V vs. SHE	0.5 M $[Bmim]BF_4$ in CH_3CN	12.1	71.2	16
Mo	-0.7 V vs. SCE	0.2 M Na_2SO_4	0.05	~55	17
Ni	-1.0 V vs. RHE	0.1 M $KHCO_3$	4.9 ± 1.3	2.3 ± 2.4	18
$Co(CO_3)_{0.5}(OH)\cdot 0.11H_2O$	-0.98 V vs. SCE	0.1 M $NaHCO_3$	0.6	97	19
BDD films[h]	-1.3 V vs. Ag/AgCl	1 M NH_3 aqueous solution	-	24.3	20
Dehydrogenases immobilized on carbon felt	-1.2 V vs. Ag/AgCl	-	~0.07	10	21

[a] Total current density of CO_2 reduction; [b] Faradaic efficiency; [c] immobilization of pyridine derivative (PYD) within the Cu-Pt alloy; [d] RuO_2/TiO_2nanotubes composite modified Pt electrode; [e] Cu_2O catalyst supported on MWCNTs functionalized with carboxyl groups; [f] Cu-containing metal-organic porous materials supported in gas diffusion electrodes; [g] Mo-Bi bimetallic chalcogenide nanosheet; [h] Si (111) wafers using a microwave plasma-assisted chemical vapor deposition system.

Table 2. Characterization results of the $Cu_{2-x}Se(y)$ nanocatalysts synthesized in mixed solvents of different V_{DETA}/V_{H2O} ratios with 15 h solvothermal times.

Catalysts	V_{DETA}: V_{H2O}	Cu(I):Cu(II) (XPS)	Cu:Se (XPS)	Tafel slope (mV dec^{-1})	C_{dl} (mF cm^{-2})	R_{ct} (Ω)
$Cu_{1.61}Se(1/0)$	1:0	3.13	1.61	149.90	9.06	219.50
$Cu_{1.60}Se(3/1)$	3:1	3.00	1.60	141.20	12.56	144.30
$Cu_{1.63}Se(1/1)$	1:1	3.41	1.63	146.50	13.40	127.90
$Cu_{1.63}Se(1/3)$	1:3	3.41	1.63	135.90	18.30	109.10
$Cu_{1.62}Se(1/5)$	1:5	3.26	1.62	139.30	14.06	116.00
$Cu_{1.64}Se(0/1)$	0:1	3.56	1.64	146.80	11.78	176.40

Table 3. Overview of reported cell voltage for CO_2 reduction in different electrolytes.

Electrode/ electrocatalysts	Electrolyte	Cell voltage (V)	Applied potential (V)	j_{tot}[a] (mA cm^{-2})	product	FE[b] (%)	References
$Cu_{1.63}Se$(1/3)	[Bmim]PF_6 (30 wt%)/CH_3CN/H_2O (5 wt%)	2.4	-2.1 V vs. Ag/Ag^+	41.5	CH_3OH	76.2±2.0	This work
Sn/GDE[c]	deionized water	3.5	-	140	HCOOH	94	22
Sn-GDE[d]	0.45 M $KHCO_3$ + 0.5 M KCl	3.7	-1.5 V vs. Ag/AgCl	150	HCOOH	70	23
Sn	catholyte-free	2.2	-	51.7	HCOOH	93.3	24
Iron porphyrin[e]	0.1 M KCl + 0.5 M $KHCO_3$	2.5	-0.96 V vs. SHE	1	CO	~90	25
Ag	0.1 M $KHCO_3$	~3.4	-1.1 V vs RHE	-	CO	~80	26
Co phtalocyanine/carbon colth	0.5 M $NaHCO_3$	2.5-3.0	-	1-6	CO	90	27
Ni single atoms/Graphene nanosheets	0.5 M $KHCO_3$	2.78	-0.7 V vs RHE	50	CO	95	28
BDD electrode	0.1M NBu_4PF_6/CH_3CN	3.0	-	-	CO	76	29

[a] Total current density of CO_2 reduction; [b] Faradaic efficiency; [c] imidazole-nanoparticle Sn catalyst-based GDE; [d] Sn nanoparticles on gas diffusion electrodes; [e] Iron porphyrin immobilized into a conductive Nafion/carbon powder.

Table 4. Total current density and Faradaic efficiency of methanol using $[Bmim]PF_6/CH_3CN/H_2O$ (5 wt%) electrolytes with different $[Bmim]PF_6$ contents over $Cu_{1.63}Se(1/3)$ nanocatalysts.

Entry	$[Bmim]PF_6$ mass fraction (%)	j_{tot}^{a}(mA cm^{-2})	$FE^{a}_{methanol}$(%)
1	5	12.4	7.1±2.2
2	10	22.1	19.3±1.8
3	15	28.0	30.1±2.1
4	20	34.7	45.7±1.6
5	30	41.5	77.6±2.4
6	40	39.8	64.2±1.2
7	50	33.0	41.5±2.3
8	60	26.1	27.1±1.9
9	70	16.2	14.5±1.5

[a] Total current density of CO_2 reduction; [b] Faradaic efficiency.

Table 5. Total current density and Faradaic efficiency of methanol using [Bmim]PF_6 (30 wt%)/CH_3CN/H_2O electrolytes with different H_2O contents over $Cu_{1.63}Se$(1/3) nanocatalysts.

Entry	H_2O mass fraction (%)	j_{tot}^{a} (mA cm^{-2})	$FE^{b}_{methanol}$ (%)
1	0	32.5	38.1±1.6
2	2.5	40.8	54.3±1.5
3	5	41.5	77.6±2.4
4	7.5	40.2	56.4±2.0
5	10	39.9	47.8±1.7
6	15	34.6	33.7±1.6
7	20	31.3	29.1±1.3

[a] Total current density of CO_2 reduction; [b] Faradaic efficiency.

The effects of ionic liquid (IL) [Bmim]PF_6 and H_2O contents was investigated on $Cu_{1.63}Se$(1/3) electrode at -2.1 V vs. Ag/Ag^+ in the ternary electrolyte. With increasing IL content in the electrolyte, the current density increased to 41.5% (30 wt% [Bmim]PF_6 content) and then decreased gradually (Supplementary Table 4). When the IL content exceeded 30 wt%, the motion of the ions was hindered and the rate of charge transfer was reduced on the electrode surface which was mainly caused by the enhanced viscosity and electrostatic attraction between the anions and cations of the [Bmim]PF_6[30]. It is also noteworthy that the selectivity of methanol was also significantly enhanced when trace amount of H_2O was added in the mixture electrolyte (Supplementary Table 5). Hence, in [Bmim]PF_6 (30 wt%)/CH_3CN/H_2O (5 wt%) ternary electrolyte, the activity of the catalysts and the production of methanol is optimal.

Table 6. ECSA and normalized $j_{methanol}$ of various catalysts.

Entry	Catalysts	ECSA (cm^{-2})	normalized $j_{methanol}$ ($mA·cm^{-2}$)
1	$Cu_{1.63}Se$(1/3)	0.96	33.5
2	Cu_2Se	0.71	9.3
3	Cu_2S	0.60	6.3
4	Cu_2O	0.51	2.2
5	CuSe	0.54	5.5
6	CuS	0.46	4.5
7	CuO	0.40	1.5
8	Cu	0.35	0.6

Table 7. Structural parameters of $Cu_{1.63}Se$(1/3), Cu_2Se and CuSe extracted from the EXAFS fitting. (S_0^2=0.80)[a].

Catalysts	Scattering pair	CN[b]	R (Å)[c]	σ^2 (10^{-3}Å^2)[d]	ΔE_0 (eV)[e]	R factor[f]
$Cu_{1.63}Se$(1/3)	Cu-Se	3.1±0.5	2.52±0.02	4.3±1.9	3.2±1.2	0.019
Cu_2Se	Cu-Se	4	2.53±0.01	7.6±0.5	3.8±0.6	0.010
CuSe	Cu-Se	6	2.51±0.03	3.9±0.7	6.5±0.9	0.010

[a] S_0^2 is the amplitude reduction factor; [b] CN is the coordination number; [c] R is interatomic distance (the bond length between central atoms and surrounding coordination atoms); [d] σ^2 is Debye-Waller factor (a measure of thermal and static disorder in absorber-scatterer distances); [e] ΔE_0 is edge-energy shift (the difference between the zero kinetic energy value of the sample and that of the theoretical model). [f] R factor is used to evaluate the goodness of the fitting.

Table 8. Methanol obtained from the electroreduction of CO_2 over $Cu_{1.63}Se(1/3)$ electrodes in the presence of possible reaction intermediates.

Entry	Reaction	Production rate (μmol/h)
1	without CO_2	-
2	CO_2 only	163.85
3[a]	HCOOH only	-
4[b]	CO only	177.20
5[c]	HCHO only	180.51
6[d]	CO_2 + HCOOH	160.17
7[e]	CO_2 + CO	175.45
8[f]	CO_2 + HCHO	183.11

[a] 1M HCOOH in the electrolyte; [b] CO stream of 5 sccm; [c] 1M HCHO in the electrolyte; [d] 1M HCOOH in the electrolyte and CO_2 stream of 5 sccm; [e] CO_2 stream of 5 sccm and CO stream of 5 sccm; [f] 1M HCHO in the electrolyte and CO_2 stream of 5 sccm.

Table 9. The correction of zero point energy, enthalpy effect, and entropy effect of the adsorbed and gaseous species.

	ZPE (eV)	$\int C_P dT$ (eV)	TS (eV)
*COOH	0.62	0.10	0.18
*CO	0.19	0.08	0.15
*CHO	0.44	0.09	0.18
*OCH_2	0.76	0.09	0.19
*OCH_3	1.11	0.09	0.18

Supplementary references

1. Yang, H.-P. *et al.* Organically doped palladium: a highly efficient catalyst for electroreduction of CO_2 to methanol. *Green Chem.* **17**, 5144-5148 (2015).
2. Zhang W. Y. et al. Electrochemical reduction of carbon dioxide to methanol on hierarchical Pd/SnO_2 nanosheets with abundant Pd-O-Sn interfaces, *Angew. Chem. Int. Ed.* **57**, 9475-9479 (2018).
3. Yang, H.-P. *et al.* Selective electrochemical reduction of CO_2 to different alcohol products by an organically doped alloy catalyst. *Green Chem.* **18**, 3216-3220 (2016).
4. Barton Cole, E. *et al.* Using a one-electron shuttle for the multielectron reduction of CO_2 to methanol: kinetic, mechanistic, and structural insights. *J. Am. Chem. Soc.* **132**, 11539-11551 (2010).
5. Lu, L. *et al.* Highly efficient electroreduction of CO_2 to methanol on palladium-copper bimetallic aerogels. *Angew. Chem. Int. Ed.* **57**, 14149-14153 (2018).
6. Lee, J. H. Q., Lauw, S. J. L. & Webster, R. D. The electrochemical reduction of carbon dioxide (CO_2) to methanol in the presence of pyridoxine (vitamin B6). *Electrochem. Comm.* **64**, 69-73 (2016).
7. Giesbrecht, P. K. & Herbert, D. E. Electrochemical reduction of carbon dioxide to methanol in the presence of benzannulated dihydropyridine additives. *ACS Energy Lett.* **2**, 549-555 (2017).
8. Qu, J. *et al.* Electrochemical reduction of CO_2 on RuO_2/TiO_2 nanotubes composite modified Pt electrode. *Electrochim. Acta* **50**, 3576-3580 (2005).
9. Popić, J. P., Avramov-Ivić, M. L. & Vuković, N. B. Reduction of carbon dioxide on ruthenium oxide and modified ruthenium oxide electrodes in 0.5 M $NaHCO_3$. *J. the Electrochem. Soc.* **421**, 105-110 (1997).
10. Malik, M. I. *et al.* Electrochemical reduction of CO_2 to methanol over MWCNTs impregnated with Cu_2O. *Chem. Eng. Sci.* **152**, 468-477 (2016).

11. Albo, J. *et al.* Production of methanol from CO_2 electroreduction at Cu_2O and Cu_2O/ZnO-based electrodes in aqueous solution. *Appl. Catal. B: Environ.* **176**, 709-717 (2015).

12. Schizodimou, A. & Kyriacou, G. Acceleration of the reduction of carbon dioxide in the presence of multivalent cations. *Electrochim. Acta* **78**, 171-176 (2012).

13. Andrews, E. *et al.* Electrochemical reduction of CO_2 at Cu nanocluster/(1010) ZnO electrodes. *J. Electrochem. Soc.* **160**, 841-846 (2013).

14. Le, M. *et al.* Electrochemical reduction of CO_2 to CH_3OH at copper oxide surfaces. *J. Electrochem. Soc.* **158**, 45-49 (2011).

15. Albo, J. *et al.* Copper-based metal-organic porous materials for CO_2 electrocatalytic reduction to alcohols. *ChemSusChem* **10**, 1100-1109 (2017).

16. Sun, X. *et al.* Molybdenum-bismuth bimetallic chalcogenide nanosheets for highly efficient electrocatalytic reduction of carbon dioxide to methanol. *Angew. Chem. Int. Ed.* **55**, 6771-6775 (2016).

17. Summers, D. P., Leach, S. & Frese, K. W. The electrochemical reduction of aqueous carbon dioxide to methanol at molybdenum electrodes with low overpotentials. *J. Electroanal. Chemistry and Interfacial Electrochem.* **205**, 219-232 (1986).

18. Kuhl, K. P. *et al.* Electrocatalytic conversion of carbon dioxide to methane and methanol on transition metal surfaces. *J. Am. Chem. Soc.* **136**, 14107-14113 (2014).

19. Huang, J. *et al.* Rethinking $Co(CO_3)_{0.5}(OH)0.11H_2O$: a new property for highly selective electrochemical reduction of carbon dioxide to methanol in aqueous solution. *Green Chem.*, **20**, 2967-2972 (2018).

20. Jiwanti, P. K. *et al.* Selective production of methanol by the electrochemical reduction of CO_2 on boron-doped diamond electrodes in aqueous ammonia solution. *RSC Adv.* **6**, 102214-102217 (2016).

21. Schlager, S. *et al.* Electrochemical reduction of carbon dioxide to methanol by direct injection of electrons

into immobilized enzymes on a modified electrode. *ChemSusChem* **9**, 631-635 (2016).

22. Yang, H. et al. Electrochemical conversion of CO_2 to formic acid utilizing Sustainion™ membranes. *J. CO_2 Util.* **20**, 208-217 (2017).

23. Del Castillo, A. *et al.* Sn nanoparticles on gas diffusion electrodes: Synthesis, characterization and use for continuous CO_2 electroreduction to formate. *J. CO_2 Util.* **18**, 222-228 (2017).

24. Lee, W. *et al.* Catholyte-free electrocatalytic CO_2 reduction to formate. *Angew. Chem. Int. Ed.* **57**, 6883-6887 (2018).

25. Tatin, A. *et al.* Efficient electrolyzer for CO_2 splitting in neutral water using earth-abundant materials. *Proc. Natl. Acad. Sci. U. S. A.* **113**, 5526-5529 (2016).

26. Vermaas, D. A. & Smith, W. A. Synergistic electrochemical CO_2 reduction and water oxidation with a bipolar membrane. *ACS Energy Lett.* **1**, 1143-1148 (2016).

27. Morlanés, N., Kazuhiro, T. & Valentin, R. Simultaneous reduction of CO_2 and splitting of H_2O by a single immobilized cobalt phthalocyanine electrocatalyst. *ACS Catal.* **6**, 3092-3095 (2016).

28. Jiang, K. *et al.* Isolated Ni single atoms in graphene nanosheets for high-performance CO_2 reduction. *Energy & Environ. Sci.* **11**, 893-903 (2018).

29. Chen, Z. *et al.* Splitting CO_2 into CO and O_2 by a single catalyst. *Proc. Natl. Acad. Sci. U. S. A.* **109**, 15606-15611 (2012).

30. Zhu, Q. *et al.* Efficient reduction of CO_2 into formic acid on a lead or tin electrode using an ionic liquid catholyte mixture. *Angew. Chem. Int. Ed.* **55**, 9012-9016 (2016).
